# Rapid Fabrication of Renewable Carbon Fibres by Plasma Arc Discharge and Their Humidity Sensing Properties

**DOI:** 10.3390/s21051911

**Published:** 2021-03-09

**Authors:** Yi Chen, Fang Fang, Robert Abbel, Meeta Patel, Kate Parker

**Affiliations:** 1 Scion, 49 Sala Street, Private Bag 3020, Rotorua 3046, New Zealand; Robert.Abbel@scionresearch.com (R.A.); Meeta.Patel@scionresearch.com (M.P.); Kate.Parker@scionresearch.com (K.P.); 2 National Isotope Centre, GNS Science, 30 Gracefield, Lower Hutt 5010, New Zealand; V.Fang@gns.cri.nz

**Keywords:** plasma arc discharge, lignin fibres, renewable carbon fibres, globular structure, humidity sensing

## Abstract

Submicron-sized carbon fibres have been attracting research interest due to their outstanding mechanical and electrical properties. However, the non-renewable resources and their complex fabrication processes limit the scalability and pose difficulties for the utilisation of these materials. Here, we investigate the use of plasma arc technology to convert renewable electrospun lignin fibres into a new kind of carbon fibre with a globular and porous microstructure. The influence of arc currents (up to 60 A) on the structural and morphological properties of as-prepared carbon fibres is discussed. Owing to the catalyst-free synthesis, high purity micro-structured carbon fibres with nanocrystalline graphitic domains are produced. Furthermore, the humidity sensing characteristics of the treated fibres at room temperature (23 °C) are demonstrated. Sensors produced from these carbon fibres exhibit good humidity response and repeatability in the range of 30% to 80% relative humidity (RH) and an excellent sensitivity (0.81/%RH) in the high RH regime (60–80%). These results demonstrate that the plasma arc technology has great potential for the development of sustainable, lignin-based carbon fibres for a broad range of application in electronics, sensors and energy storage.

## 1. Introduction

Humidity sensors have been successfully applied in various fields, such as food packaging, and healthcare, as well as industrial processes [1,2,3,4]. The humidity sensing performance is determined as the relative change of an electrical property, such as capacitance or resistance [5]. Capacitive-type humidity sensors generally make use of a changing of dielectric constant upon humidity variations, as seen for metal oxide or ceramic based humidity sensors [3]. However, their long recovery time poses limitations on practical applications. Another sensing mechanism is resistive that changes electrical conductance upon interaction with water vapour/molecules. Carbon nanomaterials such as carbon nanotubes, graphene and carbon nanofibres have recently attracted widespread research interest due to their resistive humidity sensing properties [6,7,8,9]. Carbon (nano)fibres are one-dimensional (1D) carbonaceous materials with a diameter in the range of 10 to 500 nm, and afford a high surface-to-volume ratio and advantageous electrical characteristics [10,11]. Compared to graphene and carbon nanotubes, carbon (nano)fibres are usually amorphous with randomly distributed graphite micro-crystallites and a distorted graphite layer. They contain many voids, allowing easy surface and structure modifications to create versatile materials with a wide range of functionalities and applications, such as sensors, energy storage, electronics, catalysts and separation membranes [10,11,12,13]. Our previous research results also suggest that the fibrous structure of nanofibres is beneficial for high performance humidity sensing [1]. Table 1 shows a comparison of the four main methods used for the synthesis of carbon nanofibres and their applications. Among these, the method that has the greatest potential for large-scale and ease of production is the electrospinning of a carbon precursor that is subsequently converted into carbon by thermal treatment [14]. Traditionally, petroleum-based polymers, such as polyacrylonitrile (PAN), have been widely used as the electrospinning carbon precursors [15]. However, PAN is expensive, and its preparation is associated with large greenhouse gas emissions (33.5 kg CO_2_ kg^−1^) [16]. Moreover, the carbonisation of PAN releases HCN and NH_3_, which have significant health and safety risks. Therefore, it cannot satisfy the fast-growing demand for carbon nanofibres produced in a sustainable manner.

Biomass, a renewable resource, has become an attractive alternative for producing low cost and sustainable carbonaceous materials with minimal environmental impact [22,23]. Lignin is regarded as a promising carbon precursor for submicron diameter carbon fibre production [21,24,25,26,27]. This is due to its high carbon content, polyaromatic structure and low cost as a by-product of the pulping industry. Lignin can be transformed into fibres through electrospinning, and then carbonised using thermal pyrolysis or hydrothermal methods [22,23,24]. However, both methods are relatively slow processes (at least several hours) and require either high temperature or high pressure [28]. Furthermore, to control the nano/micro structure of carbonised lignin fibres for better performance, post-treatments, such as doping and modification, are needed in most cases [11,13,29]. Therefore, it is highly desirable to develop rapid carbonisation technologies with controllable micro/nano structures that are also scalable, low-cost and easy to operate [30,31].

Plasma arc technology (also known as arc discharge technology) has emerged as a fast and environmentally friendly technique to produce high-quality carbon nanomaterials from carbon precursors on a large scale [32,33,34]. The thermal processes in the plasma arc technique provide very efficient means for producing novel materials owing to their high temperature, high enthalpy and high speed of quenching, which causes the homogenous condensation of the gas phases [35]. However, it currently either uses fossil-based carbon precursors, such as graphite and carbon black, or transition metal catalysts, such as Fe, Ni and Co, for carbon material production. Fossil-based precursors are not suitable for sustainable production. Moreover, metal catalysts can be incorporated into the carbon structures during plasma treatment and form impurities that are hard to remove. Furthermore, it has been reported that the structures of carbon nanomaterials prepared by the plasma arc technology are strongly influenced by varying the processing parameters, such as arc currents, process duration, pressure and other physical conditions that constitute the plasma arc process [35,36]. Among these parameters, arc current is the most significant parameter affecting the quality, yield and structure of the synthesised carbon nanostructures.

Here, we use the plasma arc technology with differing levels of arc current to successfully convert lignin fibres into micro-structured carbon fibres without a catalyst. The application of these carbon fibres for resistive humidity sensing was then demonstrated as a proof-of-concept. The lignin-based plasma arc technology paves the way for the catalyst-free, low-cost, scalable and fast production of high purity renewable carbon materials.

## 2. Materials and Methods

### 2.1. Material Fabrication

**Figure 1:**
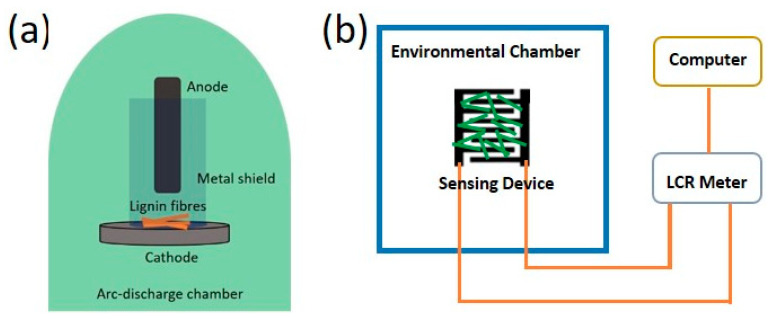
(**a**) Schematic diagram of the plasma arc discharge apparatus used to treat lignin fibres. (**b**) Schematic of the humidity sensor measurement setup.

Lignin fibres were prepared through the electrospinning of a lignin formulation following a published process [12,37]. Direct current (DC) plasma arc treatment on the lignin fibres was carried out in an arc discharge apparatus developed in-house [33,38]. Figure 1a shows the setup of the plasma arc discharge apparatus used in this work. The apparatus consists of two main parts: a pure graphite rod (Graphitestore, Northbrook, IL, USA), which serves as the anode with a diameter of 10 mm, and a water-cooled graphite disc as the cathode inside the process chamber. The anode position was controlled in the vertical direction by a stepper motor to facilitate the formation of a plasma arc with a steady current. The chamber’s partial pressure was closely monitored and maintained at 300 Torr to prevent the residual gases in the chamber from reacting with the lignin during the treatment. To study the effects of the arc current on the structure of the resulting material, different levels of current were applied in this work (10, 20, 35, 45 and 60 A). When the plasma arc was ignited between the tips of the graphite anode and the cathode, it passed through the lignin fibre sample which was placed on top of the graphite cathode disc. The discharge was maintained for 5 s at a constant current. It was difficult to exactly quantify the yield after arc discharge [39]. This was due to the fact that a certain amount of powder adhered on the inner walls of the chamber after treatment and was difficult to collect. The design of the collection setup needs to be optimised to allow all the final products to be collected after the plasma arc treatment.

### 2.2. Materials Characterisation

The morphologies of the as-obtained samples were evaluated using a JSM-6700F (JEOL, Tokyo, Japan) field-emission scanning electron microscope (FE-SEM) at a voltage of 3 kV. The samples were sputter coated with chromium (thickness of 10 nm) prior to observation. The fibre diameters of treated samples were calculated from the SEM images using ImageJ software (National Institute of Health, Bethesda, MD, USA). The reported diameters represent the averages of 50 different fibres. Fourier transform infrared (FTIR) spectra were recorded on a Nicolet FTIR 8700 spectrometer (Thermo Fisher Scientific Inc., Boston, MA, USA) in the range of 500–4000 cm^−1^ using 32 scans. Raman spectra were recorded using a Ramanstation400 instrument (PerkinElmer, Boston, MA, USA) equipped with a 785 nm (1.58 eV) excitation laser. Each spectrum was collected from a spot using five consecutive exposures to the laser (exposure time 60 s). The obtained Raman spectra were re-plotted and analysed with Origin software (OriginLab, Northampton, MA, USA) using Gaussian peak fitting. Thermogravimetric analysis (TGA) was conducted using a TA Instruments Q500 (TA Instruments, New Castle, DE, USA). The samples were heated at a rate of 10 °C min^−1^ from room temperature to 1000 °C under an air flow of 10 mL min^−1^. X-ray diffraction (XRD) patterns were obtained using an EMPYREAN diffractometer system (PANalytical, Malvern, UK) fitted with a Cu Kα X-ray tube and recorded from 2° to 90°, with a step size of 0.026°. The elemental analysis of the samples was conducted using an elemental analyser (Thermo Scientific™ FlashSmart™, Boston, MA, USA).

### 2.3. Humidity Sensor Testing

Finally, the lignin fibres treated with an arc current of 45 A were used to demonstrate a humidity sensor application (Figure 1b). The as-obtained powder was dispersed in acetone at 1 wt% and coated on interdigitated electrodes (20 mm × 14 mm, 2 mm finger width and 1 mm inter-gap spacing) that had been screen printed with silver ink (PSI-219, Novacentrix, Austin, TX, USA) on polyethylene terephthalate (PET, Officemax, New Zealand) substrate [40]. The resistive-type humidity sensing properties were tested in an EXCAL environmental chamber (Climats, Saint Médard d’Eyrans, France) with programmed relative humidity (RH) varying from 30% to 80% by changing the amount of moisture in the air, in steps of 10% RH every 15 min at a fixed temperature of 23 °C. The direct current (DC) resistance of carbon fibres under different humidity levels were measured using an inductance-capacitance-resistance (LCR) meter (IM 3536, Hioki, Nagano, Japan) under an applied voltage of 1 V [1]. The sensor response was determined using Equation (1),

(1) $$\mathrm{Sensor}\;\mathrm{response}\;\left(\%\right)=\;\left[\frac{R-R_{0}}{R_{0}}\right]\times 100$$

where *R* and *R*_0_ are the resistances at a given RH level and at 30% RH, respectively. The humidity sensor response and recovery times were measured by switching the RH from 30% RH to 80% RH and from 80% RH to 30% RH, respectively.

## 3. Results and Discussion

**Figure 2:**
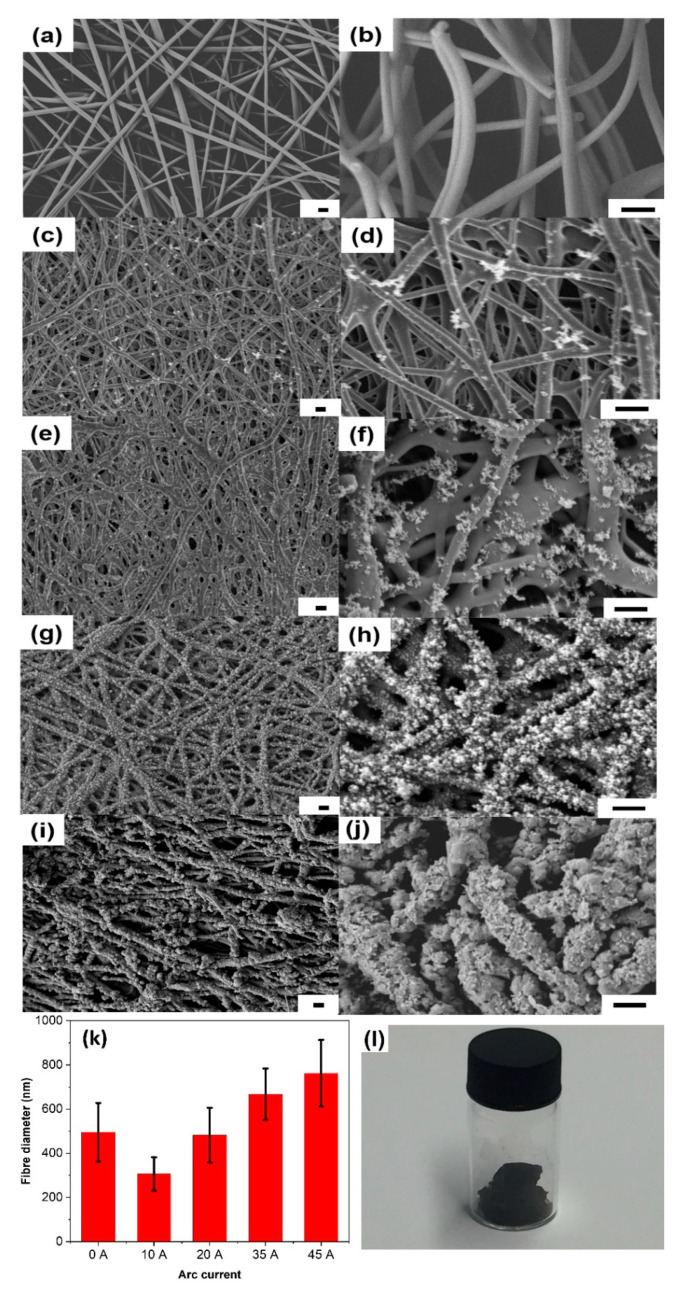
Low and high magnification SEM images of the carbon fibres treated with different levels of arc current: (**a**,**b**) 0 A (starting material; untreated lignin fibres), (**c**,**d**) 10 A, (**e**,**f**) 20 A, (**g**,**h**) 35 A, and (**i**,**j**) 45 A. All scale bars are 1 μm. (**k**) Average thicknesses of the fibres prepared with different levels of arc current. (**l**) Sample collected after arc discharge at 45 A.

Typical SEM images of the electrospun lignin fibres prior to the arc discharge treatment are shown in Figure 2a,b. These untreated lignin fibres are randomly oriented and possess diameters of approximately 500 nm. There is no evidence of beads and/or beaded fibres, indicating that the electrospinning process was successful. Upon applying an electric field between the graphite electrodes, an plasma arc was generated, and a current of electrons and plasma ions passed through the fibre network. This increased the temperature across the electrodes, resulting in the fast carbonisation of the lignin fibres. Previously, it has been reported that lignin is more promising for the rapid generation of graphitic carbon by laser treatment than cellulosic materials due to its good thermal stability [24,30]. This is supported by this work which showed the fibre-like structures were retained after the arc discharge treatment (Figure 2c–j). More interestingly, the formation of globular microstructures on the fibre surfaces was observed and this effect became more significant with higher applied currents. Statistical analysis of the fibre diameters revealed a significant decrease in thickness for samples treated at 10 A in comparison with the untreated fibres. By contrast, for higher currents (20 A and above), an upward trend in fibre thickness was seen as the current used during treatment was increased (Figure 2k). An explanation is discussed in the following paragraph. It was difficult to calculate the yield of the carbon fibres after arc discharge as only the black powder on the electrode surface was collected (Figure 2l).

The significant morphological changes induced by the plasma arc treatment are not often observed during the carbonisation of lignin fibres by slower and less vigorous methods, such as high-temperature treatment. Moreover, the arc discharge currents (up to 60 A) and treatment times (5 s) were not sufficient for the generation and deposition of nano-structured carbon from the graphite anode, which requires either higher current (i.e., 100 A) or much longer treatment times (i.e., 20 min) [35,41]. A possible explanation for the carbonisation and formation of globular structures on the fibre surfaces is the release and subsequent deposition of volatile gaseous products from the softened lignin fibre matrix by pyrolysis during arc discharge [30,42,43]. At low currents (and the associated lower temperatures), the volatiles release quite slowly and are only partially converted into carbon, then precipitated [43]. The loss of gaseous materials leads to a shrinkage of the fibres when the lignin fibres have been treated at 10 A. By contrast, the more vigorous heating at higher current levels leads to partial fibre melting and more efficient carbonisation of volatile gases. This results in the fusion of the fibres and a denser coverage of the fibre surfaces with carbonaceous precipitates that were converted from volatile gases. When the lignin fibres were treated with a higher arc current of 45 A, the formation of open porous structures was also observed (Figure 2j), which is due to the further volatilisation after carbonisation at higher temperatures [42,43]. The generation of such nano-structures on the fibres’ surfaces will increase their specific surface area, resulting in more active sites which is beneficial for applications such as sensors, supercapacitors, lithium batteries and water splitting catalysis [11]. The lignin fibres were further treated with even higher current (60 A). The SEM images (Figure S1) show that most of the fibres are fused together and the porous structure disappeared. This suggests that 45 A is the optimal current for treating lignin fibres in order to obtain porous and graphitic structures for potential applications.

**Figure 3:**
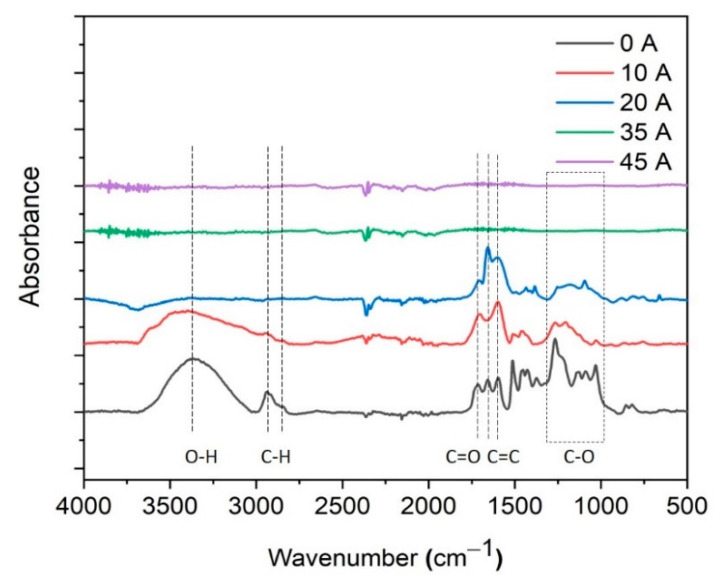
FTIR spectra of lignin and carbon fibres treated with different arc currents.

Chemical characterisation of the carbon fibres after arc discharge treatment was undertaken using FTIR (Figure 3). The spectrum of untreated lignin fibres (0 A) exhibits typical peaks corresponding to the O–H stretching at 3350–3340 cm^−1^ and C–H stretching in the methyl and methylene regions at around 2940 cm^−1^ and 2835 cm^−1^, respectively. A further band at around 1715 cm^−1^ arises from the C=O stretching in un-conjugated carbonyl/carboxyl groups [44]. The bands at 1601 cm^−1^ and 1512 cm^−1^ represent C=C aromatic ring vibrations. The peaks in the region between 1000 cm^−1^ and 1500 cm^−1^ are mainly corresponding to the C–O stretching of guaiacyl units in lignin. In the FTIR spectra of the arc discharge-treated lignin fibres, the main peaks of lignin’s functional groups become gradually weaker with increasing arc current and completely disappear in the samples treated with 35 and 45 A, indicating the full conversion of the lignin into pure carbon.

**Figure 4:**
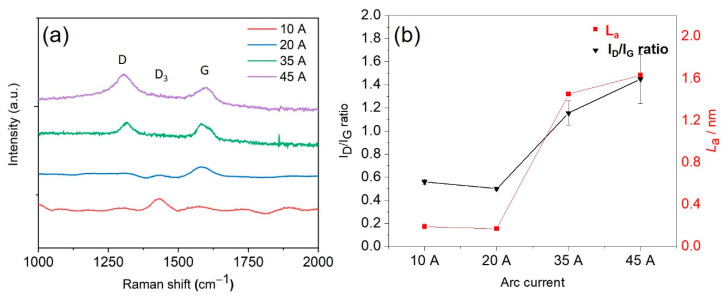
(**a**) Raman spectra of lignin fibres treated with different arc currents. (**b**) Dependence of I_D_/I_G_ ratio and graphite crystallite size (*L_a_*) on arc currents.

Raman spectroscopy was used to further characterise the structures of the carbonised fibres (Figure 4a). A peak, centred at 1430 cm^−1^ (D_3_ band, a feature of amorphous carbon), appeared in all samples treated with currents below 35 A [45]. The intensity decrease in the D_3_ band with increasingly vigorous plasma conditions confirmed the reduction in the amorphous carbon content and an increase in carbon crystallinity. The D band at 1310 cm^−1^ (disordered carbon structure) and the G band at 1590 cm^−1^ (corresponding to graphite planes) are evident for the samples treated with 35 A and 45 A. Moreover, there was a shift of the G band to higher wavenumbers, which can be attributed to an increase in the fraction of sp^2^-bonded carbons formed at high temperatures [45]. The intensity ratio of the D band to the G band (I_D_/I_G_) was measured to quantify the relative abundance of the various carbon forms in the as-obtained carbon fibres. As shown in Figure 4b, the I_D_/I_G_ ratios clearly increase with increasing arc current, from 0.56 at 10 A to 1.45 at 45 A, which is further evidence for the transformation of amorphous carbon structures into crystalline graphitic carbon domains [20,46]. The average size of the graphite crystallites (*L_a_*) has a significant positive effect on the electrical conductivities of carbon fibres [19,20], and was evaluated using Equation (2) [47],

(2) $$L_{a}^{2}\left({\mathrm{nm}}^{2}\right)=\;5.4\;\times \;10^{-2}E_{l}^{4}\left({\mathrm{eV}}^{4}\right)\frac{I_{D}}{I_{G}}$$

where *E_l_* is the laser excitation energy used for Raman spectroscopy. The *L_a_* values reveal that the lignin fibres treated at 10 and 20 A have a similar crystallite size and significantly increased from 0.63 nm to 1.45 and 1.63 nm for the samples treated at 35 and 45 A, respectively (Figure 4b). This indicates that the structure of lignin can transform into nanocrystalline graphite by arc discharge treatment at currents above 35 A.

**Figure 5:**
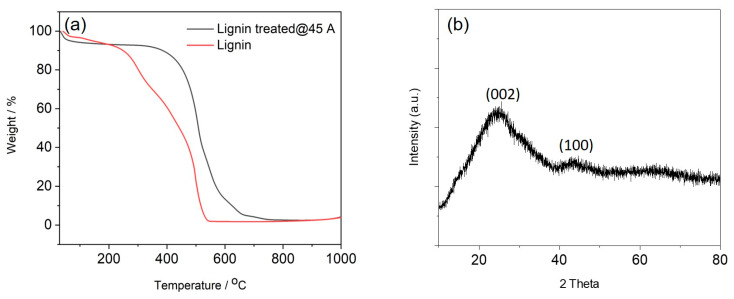
(**a**) Thermogravimetric analysis (TGA) results of lignin fibres and fibres treated with 45 A arc current, (**b**) XRD pattern of carbon fibres produced with 45 A arc current.

The thermal stability and purity of the carbon fibres are also important to ensure their high performance [32,48]. The carbon fibres treated at 45 A, which have the largest crystallite size, were further assessed with TGA in air, together with the untreated starting material (Figure 5a). The weight loss below 100 °C of both untreated lignin and carbon fibres is attributed to the loss of adsorbed water. In the temperature range between 100 and 500 °C, the untreated lignin fibres exhibited a two-step degradation due to the thermal stability differences in their oxygen-containing functional groups [49]. In contrast, the TGA result of fibres treated at 45 A showed that the degradation/oxidation only started at temperatures above 400 °C in air, indicating an improved oxidation resistance. The weight loss (around 36%) of fibres when heated from 400 to 500 °C was attributed to the decomposition of non-graphitic carbon while the weight loss between 500 and 750 °C (around 58%) was assigned to the burning of graphitic carbon domains [29,32]. This suggests that the treated fibres contain both amorphous and graphitic carbon domains. No detectable residue remained after heating above 750 °C in open air conditions, indicating the high purity of the carbon materials. Elemental composition results were further used to confirm the carbonisation of the lignin fibres (Table 2). The carbon content increased from 59.08 wt% for the untreated lignin fibres to 96.32 wt% for the arc discharge treated fibres (45 A). The hydrogen content reduced from 6.20 wt% for untreated fibres to less than 0.3 wt% for the treated fibre while the oxygen content reduced from 33.60 wt% for untreated fibres to less than 3.7 wt% for the treated fibres. The results indicate that the arc discharge treatment of lignin fibres yields fibres that are rich in carbon.

XRD was used to confirm the crystalline structure of the carbon fibres prepared at 45 A (Figure 5b). The diffraction peaks centred at 24.4° and 43.7° were assigned to the crystallographic (002) and (100) planes in the graphitic structure [29,32]. The peaks are broad, suggesting that the degree of graphitisation is still relatively low. Moreover, the interlayer spacing *d*_002_ was calculated from Bragg’s equation and found to be 0.36 nm, which is larger than that of graphite (0.335 nm). This suggests that the arc discharge treatment introduced a defective/porous structure to the fibres. The (100) crystallite size *L_c_* was calculated using the Debye formula and the value was 7.1 nm, which is similar to the *L_c_* of lignin fibres thermally carbonised at 1000 °C [50].

**Figure 6:**
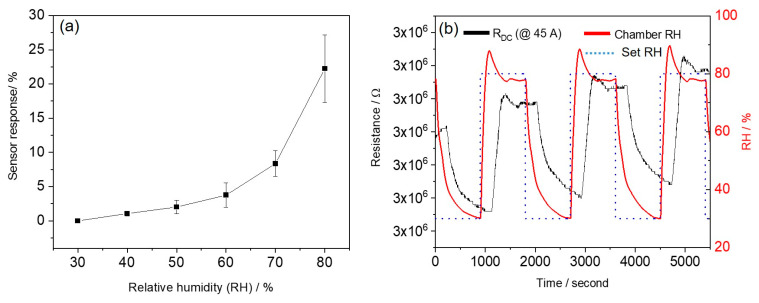
(**a**) Response of a humidity sensor based on carbon fibres (45 A) as a function of relative humidity; (**b**) dynamic response and recovery of this sensor during cycling between 30% and 80% relative humidity (RH).

The humidity sensing properties of our carbon fibres were measured at different RH levels between 30% and 80%. The fibres treated at 45 A exhibited the highest carbonisation degrees and, therefore, the highest conductivity (lowest resistance). Together with their porous structure, this renders them the best candidates for high performance sensing materials. Therefore, we exclusively used the fibres treated at 45 A to demonstrate humidity sensing in the air. As shown in Figure 6, the relative resistance of the treated fibres increased with increasing RH level, which is mainly due to the physical adsorption of water molecules on the carbon fibre surface. The electrical conduction of p-type carbon fibres is dominated by holes. With increasing humidity, water molecules are adsorbed on the carbon fibre surface that serve as electron donors. The donated electrons from the water molecular transfer to the valence band of carbon fibres, leading to a decrease in the hole concentration and increasing the electrical resistance of the carbon fibres [6,9,51]. The microporous structure of the fibres after arc discharge treatment increased the interaction surface area between water molecules and carbon fibres. This facilitates the moisture adsorption onto the fibre surface [1,6]. The specific surface area and porosity of treated fibres will be further characterised by nitrogen adsorption/desorption and Brunauer–Emmett–Teller (BET) analysis [6]. More importantly, the humidity sensitivity (0.81/%RH, defined as the slope of linearly fitted humidity response vs. RH) is greater at higher relative humidity (i.e., 60–80%). One possible reason for this is that more water molecules are adsorbed on the fibre surface at higher RH compared to the water adsorption at lower RH. Another possible reason is the difference in electron transport mechanisms between the moisture and carbon fibre sensing layer [5]. To test the sensor response and recovery, the RH in the chamber was cycled between 30% and 80% RH, at a constant temperature of 23 °C. Our previous work found that the actual humidity changes between two different RH levels (i.e., 30% and 80%) in the environmental chamber do not occur immediately due to the large volume (400 L) of the chamber [1]. Each step therefore lasted 15 min to allow for full response/recovery. When the RH was repeatedly cycled between 30% and 80%, our carbon fibre-based sensor showed a stable and reproducible response (Figure 6b).

It is evident that the resistance baseline gradually increased due to the accumulation of trapped water molecules on the carbon surface. One way to solve this problem is to heat the sensor to evaporate the adsorbed moisture [7]. In comparison with other reported carbon-based humidity sensors, such as multiwall carbon nanotubes (MWCNTs) and porous graphene (Table 3), our sensors exhibit a stronger humidity response for RH between 30% and 80%, which can be attributed to the globular and porous structure of carbon fibres induced by the plasma arc [6]. Further studies on the effects of temperature and interfering gases on the humidity sensitivity are necessary to fully understand the suitability of our sensor for potential applications in human breathing monitoring or food spoilage detection [2,4,9].

## 4. Conclusions

We have successfully fabricated micro-structured carbon fibres from electrospun lignin fibres through plasma arc treatment. After 5 s of plasma arc treatment at 45 A, the electrospun lignin fibres were transformed into carbon fibres with globular structures on their surfaces. SEM and Raman results confirmed that the structure of the carbon fibres can be controlled by using different levels of an arc current, and the graphitic crystallite size in the carbonised fibres also increased with increasing current. Further development and optimisation of the electrodes and collection setup design will enable the fast (few seconds) and large-scale production of carbon fibres by the plasma arc technique. Moreover, this method is safe and inexpensive compared to other techniques such as thermal pyrolysis or hydrothermal methods. The plasma arc discharge-induced carbon fibres were demonstrated as a humidity sensing material with high response and repeatability in the range from 30% to 80% RH. Further work is required to fully understand the ability of the sensor for potential applications. This work provides a strategy to fabricate sustainable carbon fibres with a controllable structure and crystallite size from lignin for applications in cost-efficient sensors and electronic devices.

## Figures and Tables

**Table 1:** Comparison of the main carbon nanofibre synthesis methods and applications.

Methods	Applications	Advantages	Disadvantages	Ref.
Chemical Vapour Deposition	Batteries Supercapacitors Fuel cells Sensors	High yield High performance Precise control	High energy consumption High cost	[10,17]
Template Support Growth	Batteries Supercapacitors Sensors	Controllable structure Simple operation	Limited template Extra step to remove template	[10,11]
Hydrothermal	Adsorption Oxygen reduction Supercapacitors	Simple operation High yield	High energy consumption High cost Demands of reactant Hard to control reaction	[10,11,18]
Electrospinning	Solar cells Fuel cells Batteries Supercapacitors Sensors	Low energy consumption Easy to build and operate Controllable structures and functions	Requirement post-treatment Long processing time	[10,11,19,20,21]

**Table 2:** Elemental analysis of untreated lignin fibre and arc-discharged-treated fibre (at 45 A).

Material	wt% C	wt% H	wt% N	wt% S	wt% O *
Lignin fibres	59.08	6.20	0.61	0.47	33.64
Arc-discharge-treated carbon fibres (at 45 A)	96.32	<0.3	<0.3	<0.3	<3.68

* Oxygen wt% was calculated by difference based on the other elements’ results.

**Table 3:** Comparison of the humidity sensing performance of different materials.

Sensing Materials	Output Signal	RH Range	Sensor Response	Sensitivity (%RH)	Response/Recovery Time (Minute)	Ref.
MWCNTs	Resistance	70–90%	~100% (∆*R*/*R*_0_)	Not reported	Not reported/120	[8]
Oxidised-MWCNTs	Current	33–95%	18–33% (∆*I*/*I*_0_)	0.41	5~8/7~11	[51]
Carbon nanocoil	Resistance	4–80%	~12.2% (∆*R*/*R*_0_)	0.15	0.03/0.025	[6]
Porous graphene	Resistance	12–97%	5% (∆*R*/*R*_0_)	0.022	1/7	[9]
Arc-treated carbon fibre	Resistance	30–80%	23% (∆*R*/*R*_0_)	0.08 (30–60%) 0.81 (60–80%)	3–4/3–4	This work

## Data Availability

Data availability on request.

## Supplementary Materials

The following are available online at https://www.mdpi.com/1424-8220/21/5/1911/s1, Figure S1: Low and high magnification SEM images of lignin fibres treated with 60 A.
