# Supplementary material for: Rapid Fabrication of Renewable Carbon Fibres by Plasma Arc Discharge and Their Humidity Sensing Properties
Source: Sensors (Basel). 2021 Mar 9;21(5):1911. doi: 10.3390/s21051911 (PMC7967239; doi:10.3390/s21051911)
Supplement: Supplementary file 1 [file sensors-21-01911-s001.pdf]

Supplementary Materials

Rapid Fabrication of Renewable Carbon Fibres by Plasma Arc Discharge and Their Humidity Sensing Properties

Yi Chen [1,*], Fang Fang [2], Robert Abbel [1], Meeta Patel [1] and Kate Parker [1]

[1] Scion, 49 Sala Street, Private Bag 3020, Rotorua 3046, New Zealand; Robert.Abbel@scionresearch.com (R.A.); Meeta.Patel@scionresearch.com (M.P.); Kate.Parker@scionresearch.com (K.P.)
[2] National Isotope Centre, GNS Science, 30 Gracefield, Lower Hutt 5010, New Zealand; V.Fang@gns.cri.nz
* Correspondence: yi.chen@scionresearch.com (Y.C.)

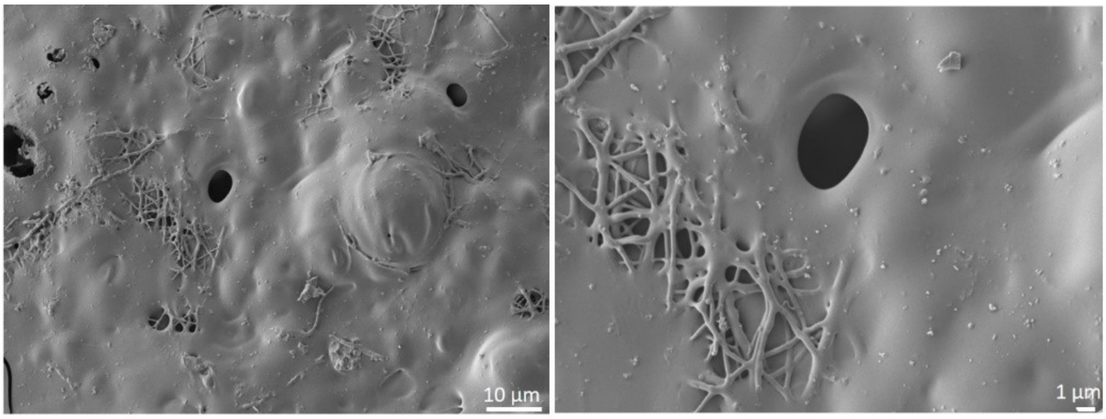

Figure S1. Low and high magnification SEM images of lignin fibres treated with 60 A.
